# A Blockchain-Based Traceability Model for Grain and Oil Food Supply Chain

**DOI:** 10.3390/foods12173235

**Published:** 2023-08-28

**Authors:** Yuan Zhang, Xuyang Wu, Hongyi Ge, Yuying Jiang, Zhenyu Sun, Xiaodi Ji, Zhiyuan Jia, Guangyuan Cui

**Affiliations:** 1 Key Laboratory of Grain Information Processing & Control, Ministry of Education, Henan University of Technology, Zhengzhou 450001, Chinajiangyuying11@163.com (Y.J.);; 2 Henan Provincial Key Laboratory of Grain Photoelectric Detection and Control, Zhengzhou 450001, China; 3 College of Information Science and Engineering, Henan University of Technology, Zhengzhou 450001, China; 4 School of Artificial Intelligence and Big Data, Henan University of Technology, Zhengzhou 450001, China

**Keywords:** blockchain, grain and oil food, supply chain, machine learning, data recovery

## Abstract

The structure of the grain-and-oil-food-supply chain has the characteristics of complexity, cross-regionality, a long cycle, and numerous participants, making it difficult to maintain the safety of supply. In recent years, some phenomena have emerged in the field of grain procurement and sale, such as topping the new with the old, rotating grains, the pressure of grades and prices, and counterfeit oil food, which have seriously threatened grain-and-oil-food security. Blockchain technology has the advantage of decentralization and non-tampering Therefore, this study analyzes the characteristics of traceability data in the grain-and-oil-food-supply chain, and presents a blockchain-based traceability model for the grain-and-oil-food-supply chain. Firstly, a new method combining blockchain and machine learning is proposed to enhance the authenticity and reliability of blockchain source data by constructing anomalous data-processing models. In addition, a lightweight blockchain-storage method and a data-recovery mechanism are proposed to reduce the pressure on supply-chain-data storage and improve fault tolerance. The results indicate that the average query delay of public data is 0.42 s, the average query delay of private data is 0.88 s, and the average data-recovery delay is 1.2 s. Finally, a blockchain-based grain-and-oil-food-supply-chain traceability system is designed and built using Hyperledger Fabric. Compared with the existing grain-and-oil-food-supply chain, the model constructed achieves multi-source heterogeneous data uploading, lightweight storage, data recovery, and traceability in the supply chain, which are of great significance for ensuring the safety of grain-and-oil food in China.

## 1. Introduction

China is a major agricultural country. Therefore, grain, as an important branch of agriculture, is an essential source of nutrition for the daily lives of its people. Ensuring grain security has always been a top priority for national security [1,2]. Grain is an important food source of minerals, dietary fiber, and other nutrients, and oily foods provide fatty acids needed by the human body [3,4]. In recent years, rice with excessive arsenic content and lead-contaminated wheat have occasionally been found in grain-quality assessments. In the field of grain purchasing and sales, there are also problems such as “increasing surplus grain”, “rotating grain”, “ topping the nw with the old”, and pressure on grades and prices [5,6]. These problems seriously affect consumer trust, which generate widespread attention. Compared with other supply chains, the grain-and-oil-food-supply chain has the characteristics of cross regionality, a long cycle, multiple participants, and heterogeneous data sources [7]. The traceability of traditional grain-and-oil-food-supply chains cannot guarantee the accuracy of data, and there are problems such as data centralization, data tampering, and asymmetric traceability information. Moreover, each link connecting the supply chain is relatively independent, and information exchange between each link is relatively difficult. Once there is a quality and safety problem, the subject of responsibility cannot be confirmed promptly [8,9]. In addition, traceability data in the grain-and-oil-food-supply chain also presents some significant challenges in terms of storage. Therefore, there is an urgent need for an advanced traceability technology to be applied to the field of grain-and-oil-food traceability to ensure the safety of grain and oil foods.

Blockchain technology is an interdisciplinary innovation technology. Its essence is a distributed database system, which is called “the next trust cornerstone” [10]. Currently, blockchain technology is widely applied in many fields, including financial services, medical-data security, and agricultural-product traceability [11,12]. Blockchain technology can simplify supply-chain processes, reduce unnecessary costs, effectively improve many pressure points in traditional supply chains, and empower supply-chain management [13]. In the field of traceability, blockchain technology has specific advantages. Its features, such as decentralization and tamper resistance, can effectively solve the problems of data centralization and easy tampering in traditional traceability [14]. The integration of blockchain technology and Internet of Things (IoT) technology into supply-chain traceability is a current trend [15]. Sensor technology can quickly collect data, such as temperature, humidity, and air pressure during the growth process of grains, without manual interference. The collected data are uploaded to the supply chain to ensure the transparency of grain-growth information. Radio-frequency identification (RFID) is a technology that has been used to identify items such as goods, foods, and books [16]. This RFID technology can quickly and effectively transmit traceability data, enabling the monitoring of the entire food chain from farmland to the dining table. Although the use of IoT devices can improve the efficiency of information collection in the supply chain, there are also certain limitations. The IoT equipment is vulnerable to the external environment and the equipment itself, which may generate abnormal data during the data-collection process, and cannot guarantee the accuracy of the source data. If the traceability data of the grain-and-oil-food-supply chain are incorrect from the source, the security of the entire supply chain is affected. In addition, blockchain technology itself has storage-performance limitations, since each node in the chain needs to store complete traceability data, which can easily create storage bottlenecks. Once traceability data are lost, there may be situations in which they cannot be retrieved or restored in a timely manner.

Zhang et al. [17] proposed a lightweight blockchain-technology data-storage architecture that can efficiently and securely store massive crop-breeding data by using proxy encryption. Peng et al. [18] built a dynamic supervision model suited to the circulation characteristics of the rice supply chain and designed three types of smart contract to ensure the quality and safety of rice. Li et al. [19] proposed a grain-food blockchain-traceability information-management model based on the master–slave multichain structure. The results showed that the grain-traceability system has certain advantages over the blockchain-single-chain structure in all respects. Zhang et al. [20] proposed a secure and trustworthy agricultural-product-traceability system, which was supported by blockchain and CP-ABE encryption technology. It can set access-control policies through data attributes and encrypt data on the blockchain. Violino et al. [21] built a traceability system for extra-virgin olive oil (EVOO) by applying RFID and QR code technology, and verified its economic sustainability. Xu et al. [22] constructed a reliable grain-and-oil-quality-and-safety-traceability model and developed a wheat-quality-and-safety-traceabilityprototype system. The results showed that the security and full-process traceability of cross-chain information interaction are guaranteed.

In order to solve the problems of data centralization and tampering in the traditional grain-and-oil-food-supply chain, blockchain technology is used to grain-and-oil-food traceability and build a grain-and-oil-food-traceability model based. On this basis, a new method combining blockchain technology and machine learning is proposed to process the source data of the supply chain with outliers to ensure the authenticity and reliability of the source data. In addition, to address the issue of redundant data storage in the grain-and-oil-food-supply chain, a lightweight blockchain storage method and data-recovery mechanism are proposed to alleviate the pressure on supply-chain-data storage and improve fault tolerance. The results show that the average query latency was 0.42 s for public data, 0.88 s for private data, and 1.2 s for average data-recovery latency. Finally, A grain-and-oil-food-supply-chain system based on blockchain is designed and built, which is of great significance to ensure the security of the grain-and-oil-food-supply chain.

## 2. Materials and Methods

### 2.1. Blockchain

**Figure 1:**
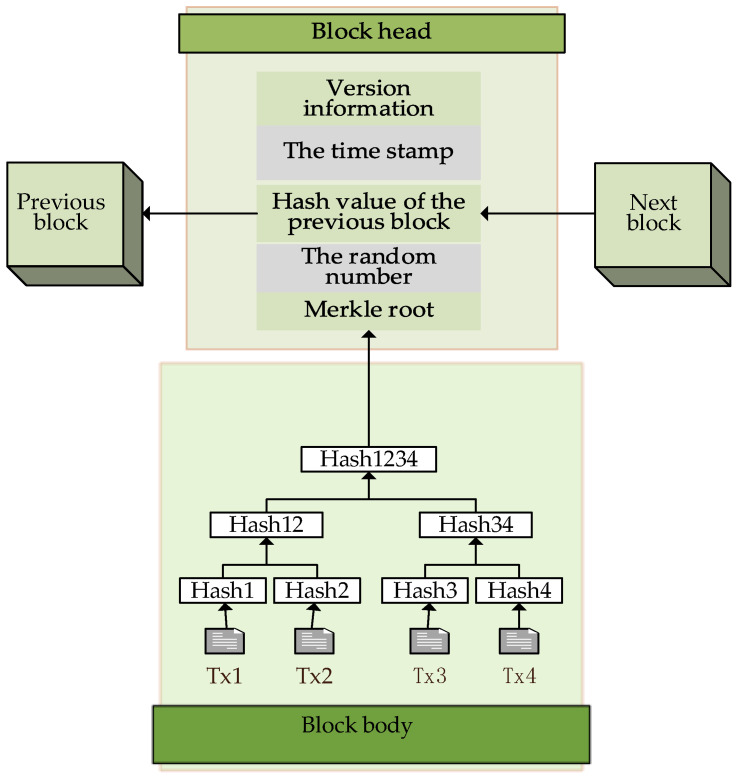
Block-structure diagram.

The basic architecture of blockchain is composed of chain blocks with time stamps, as shown in Figure 1, which suggests strong traceability. Key technologies of blockchain include distributed storage, smart contracts, consensus mechanisms, etc. Recently, some research developments have been made in the application of key technologies of blockchain to establish traceability system in grain-and-oil-food-supply chain, which can fundamentally solve the problems of traditional traceability-data centralization, easy tampering, and information islands.

Blockchain has the characteristics of distributed storage, and when it is applied to the traceability of grain-and-oil-food-supply chains, there is a problem of high data-storage pressure [23]. Therefore, it is necessary to optimize blockchain storage and alleviate storage pressure. Current storage-optimization methods include sharding technology, multi-chain architecture, and combination with interplanetary file system (IPFS) [24]. Smart contract is an important component of the contract layer in the blockchain architecture, which is a piece of code stored in the blockchain. Once the pre-set terms in the contract are triggered, the program is automatically executed without human intervention [25]. The current applications of blockchain-based smart contracts in the supply chain include contract security, smart contract design, and transaction-concurrency-execution efficiency. The consensus mechanism is the core of the entire blockchain, ensuring that each node confirms system consistency in a distributed environment and determines the operational efficiency of the entire blockchain [26]. So far, many scholars have optimized the grain-supply-chain-consensus algorithm based on blockchain from the perspective of consensus mechanisms, thus improving the traceability efficiency of the whole supply chain. Furthermore, advances have also occurred in privacy protection and access control in the blockchain-based grain-and-oil-food-supply chain. In particular, access-control research mainly includes the formulation of user-access permissions, the design and implementation of fine-grained access control, and the protection of sensitive data in the supply chain; privacy protection mainly includes identity-privacy protection and data-privacy protection, among which the application of mixed currency technology and various encryption algorithms has become a trend in supply-chain-privacy protection [27].

### 2.2. Blockchain and Internet of Things

In the traceability of grain-and-oil-food-supply chain, it is common to combine blockchain technology with Internet of Things technology. Internet of Things technology can reduce human interventions in the collection of traceability-data information and improve traceability efficiency. Forms of IoT technology, such as sensor technology, RFID, etc., are currently applied in the supply chain [28]. A novel BIOT (blockchain and the Internet of Things)-based layered framework using EOSIO has been proposed for effective food traceability [29]. The EOSIO 2.0 is a form of open-source blockchain software, which provides developers with a high-performance, scalable, and easy-to-use blockchain-application platform.

### 2.3. Machine Learning

Machine learning is the core of artificial intelligence and an interdisciplinary area that integrates multiple fields. It has been widely applied in many fields, including data mining and analysis, pattern recognition, and so on. The outlier-detection model constructed in this paper is applied to isolated forest [30], random forest [31], and logistic regression algorithm [32] in machine learning.

The first classifier of the outlier-detection model designed in this paper uses the isolated forest algorithm to detect outliers. Isolated forest is a decision-tree-based algorithm that differs from traditional clustering-classification methods, such as k-means and DBSCAN, with the goal of isolating abnormal data in samples [33,34]. The idea behind it is to use a random hyperplane to cut a data space, which can be divided into two subspaces once, and then continue to randomly select a hyperplane to cut the two subspaces of the first cut and continue to cycle until each subspace contains only one data point. The isolated forest algorithm has linear time complexity, which can effectively reduce the time cost and improve the efficiency of data processing.

The second layer is the random forest algorithm used in the design model. Random forest is an integrated algorithm. The algorithm idea is trained by multiple weak classifiers and, eventually, a strong classifier is obtained, which ensures the algorithm has high accuracy and good generalization ability.

The third layer uses logistic regression as the final decision maker. Logistic regression is often used in the second classification problem. The idea underlying it is to first fit the decision boundary, and then establish the probability relationship between the boundary and the classification, so as to obtain the probability value of the corresponding category. It has the advantages of fast classification speed and strong interpretability.

## 3. Results and Discussion

### 3.1. Grain-and-Oil-Food-Supply-Chain Model

**Figure 2:**
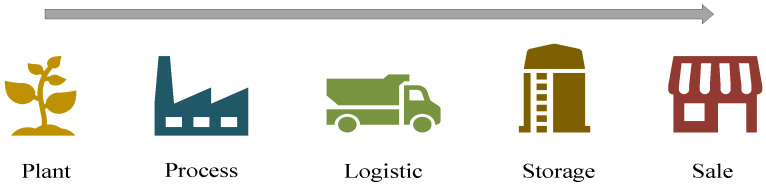
Grain-and-oil-food-supply chain.

The grain-and-oil-food-supply chain can be divided into five stages, production, processing, logistics, warehousing, and sales, as shown in Figure 2. The production stage refers to the production activities carried out by farmers on a farm, recording information on the origins of seeds, cultivators, temperature and humidity of grain during growth, and yield and quality of grain at maturity in the block. The processing stage is the process of converting raw materials into finished products, involving traceability information, such as processing equipment, processing time, batches, etc. At this stage, information including the production date and the list of raw materials used can be recorded through RFID, and a unique code can be generated. Traceability information, such as vehicle information, transportation time, and ambient temperature inside the vehicle are uploaded to the blockchain in the logistics phase. During the warehousing stage, traceability information including warehouse number, product source, and warehouse temperature need to be recorded and saved. The sales phase includes traceability data, such as store information, store-manager information, and business-license information. To ensure the sharing of traceability information while protecting the security of private data, grain-and-oil-food-related blockchain traceability data are divided into public data and private data, as shown in Table 1.

**Figure 3:**
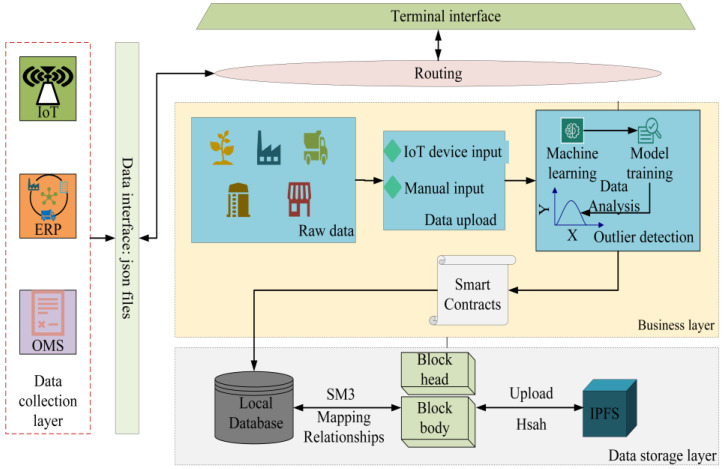
Blockchain-based trustworthy and traceable system framework for grain-and-oil-food-supply chain.

**Figure 4:**
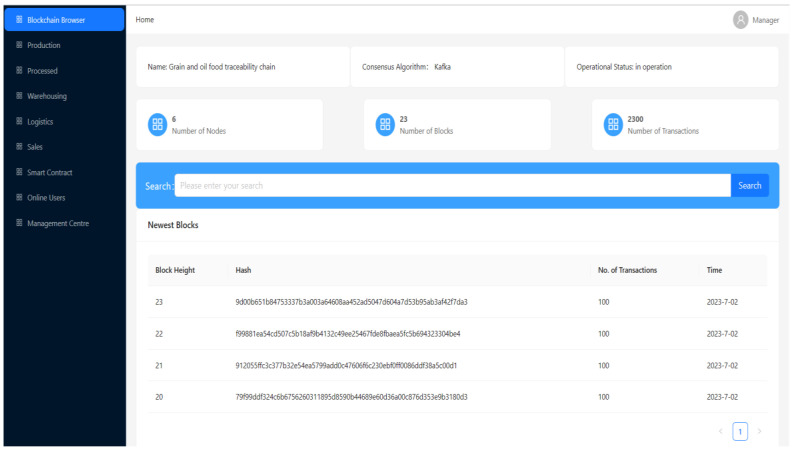
Main interface of grain-and-oil-food-traceability system.

**Figure 5:**
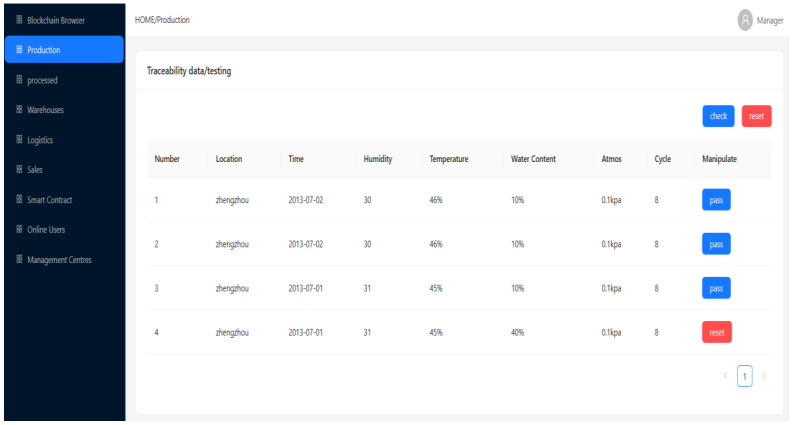
Production-stage traceability-data interface.

The system’s framework is based on Hyperledger Fabric 1.2.0, and the grain-and-oil-food-outlier-detection experiment involved is implemented using Python 3.8. Hyperledger Fabric is an open-source distributed-ledger platform designed to provide enterprises with a scalable, flexible, and secure solution. Python 3.8 is a version of the Python language, which provides Python developers with a better development experience. The computer-hardware configuration used in the experiment is as follows: the processor is 12th Gen Intel (R) Core (TM) i5-12490F 3.00 GHz, the running memory is 16 GB, and the hard drive is 1 TB. The entire system is built in the Windows 10 system, and the basic experimental environment is shown in Table 2. The main IoT technology used is RFID; in the field of traceability in the grain-and-oil-food-supply chain, RFID is applied to various links in the supply chain. The system is divided into three modules, as shown in Figure 3, where the SM3 [35] and IPFS are applied in the storage layer. The SM3 algorithm is a hash algorithm based on group iteration, released by the National Password Administration on 17 December 2010. The SM3 algorithm has high security and anti-attack ability, and can be widely used in digital signatures, identity authentication, certificate-based encryption, and other data-integrity-verification occasions. The IPFS is a new distributed file system, which can effectively solve the reliability, security, and privacy problems in the existing centralized storage system. It adopts a decentralized and peer-to-peer approach to store and access files, enabling data to be more securely stored on computer nodes around the world, and ensuring the immutability and non-=forgeability of data. The developed traceability system for grain and oil foods is shown in Figure 4 and Figure 5.

(1) Data-collection layer. In this layer, traceability data for each stage of the grain-and-oil-food-supply chain are collected through connected IoT devices (temperature and humidity sensors, RFID, etc.) and transmitted to the business layer through a unified data-exchange interface. Devices using IoT can collect and transmit temperature, humidity, geographic location, etc. Additionally, RFID tags are used to automatically identify and trace grain-and-oil-food information. These data can be shared after they are uploaded to the blockchain.

(2) Business layer. This layer includes modules such as traceability-data upload, data-outlier detection, etc. The traceability data in the whole grain-and-oil-food-supply chain are automatically or manually input into the outlier-detection module through the IoT device for data verification. If the data are normal, they are uploaded to the blockchain through the smart contract. If there are abnormal data, they are collected again in a timely manner. The characteristics of blockchain itself ensure that traceability data are open, transparent, tamper-resistant, and traceable during storage, access, and other processes. In addition, the application of consensus mechanism can also improve the traceability efficiency of the entire system, to a certain extent.

(3) Data-storage layer. In this layer, the main implementation is to store grain-and-oil-food-traceability data and establish a data-recovery mechanism to improve the system’s fault tolerance. Firstly, the traceable data are stored in the local MySQL database. Next, the SM3 algorithm is used to upload the encrypted summary and traceable private data to the blockchain to establish an index relationship with the local database. Secondly, the headers and bodies of each block are stored separately. The block bodies are uploaded to IPFS, and the returned hash values are stored in the blockchain network. The IPFS is a peer-to-peer hypermedia protocol that makes networks scalable, resilient, and interoperable [36]. At this point, the blockchain only needs to store the summary returned by IPFS, which can greatly reduce the storage pressure on the blockchain; when local data are lost or damaged, accessing IPFS through hash values stored in the blockchain can obtain complete traceability data, and the integrity of the data can be verified through the SM3 encryption algorithm, which establishes a mechanism for grain-and-oil-food-supply-chain data recovery.

### 3.2. Traceability-Data-Outlier Detection

The participants in the grain-and-oil-food-supply chain include farmers, processors, transporters, warehousing enterprises, etc. Key traceability information for each stage of the supply chain is provided in Section 3 of this paper. These data are collected through the data-collection layer, and filtered through data-anomaly detection in the business layer. If the detected datum is an outlier, it needs to be collected again in time, and the correct data are uploaded to the data-storage layer through the smart contract. The design of smart contracts includes two aspects: data-linking and tracing-data queries. The design of smart contracts is shown in Table 3.

**Figure 6:**
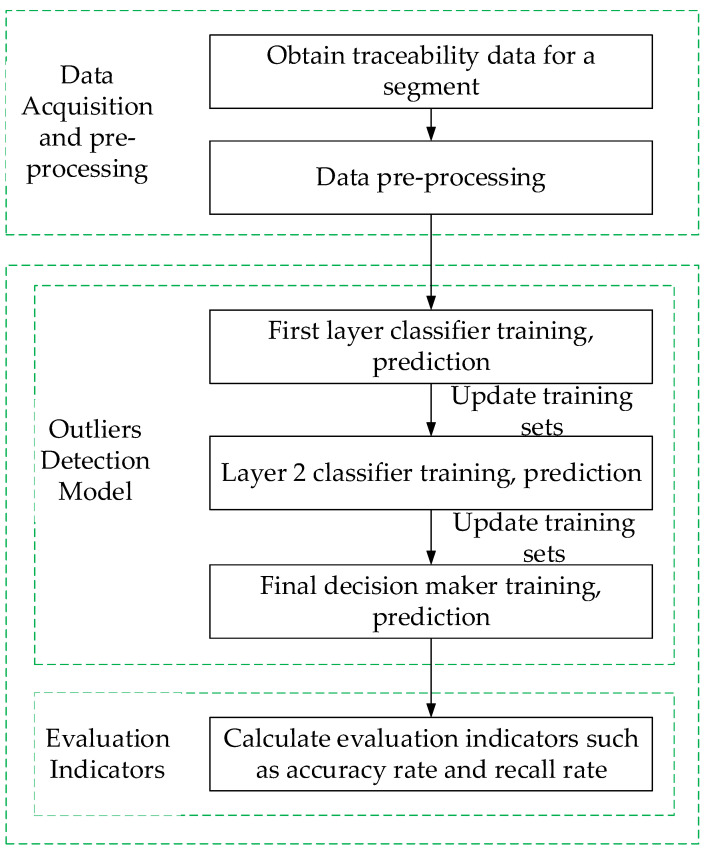
Outlier-detection process.

In order to achieve high-precision outlier detection with grain-and-oil-food-supply-chain traceability data and solve the limitations of traditional single-model detection, in this paper, the idea of ensemble learning in machine learning is used to build a three-layer anomalous-data-detection model. The first layer classifier is an isolated forest, and the second layer classifier is a random forest. In this layer, the training set needs to be updated, and the first layer’s basic classifier classifies incorrect data and correctly classified data in a 1:1 ratio. This layer is the fundamental step in the entire outlier-detection model; intensive training is carried out for the data that are incorrectly classified on the previous level to improve the accuracy of the model. The third-level logistic-regression algorithm is the final decision maker, fully leveraging the advantages of different classifiers. The overall process of the proposed anomaly detection model is shown in Figure 6.

The outlier-detection problem can be regarded as a two-classification problem and, usually, the confusion matrix is used to record these classifications, as shown in Table 4. The detection of abnormal data in grain-and-oil-food traceability can be regarded as a binary classification problem. In this paper, the evaluation criteria for outlier-detection models are precision rate, recall rate, and F1 score, as shown in Table 5. The F1 value is a comprehensive evaluation index of the accuracy and recall rate. The higher the F1 value, the better the detection performance of abnormal values for grain and oil foods.

The test data used in this paper are the planting-process data of the grain-and-oil-food-supply chain provided by a specific company. There are a total of one thousand data sets, each containing nine characteristics, such as growth temperature, humidity, and soil moisture. The dataset is randomly divided into a training set and a testing set, with 700 training sets, 70 abnormal data, 300 testing sets, and 30 abnormal data.

In the outlier-detection model designed in this paper, the first-layer classifier and the second-layer classifier are isolated forest and random forest, respectively. The two models were compared with the model proposed in this paper, and the detection effect is shown in Table 6.

In Table 6, it can be seen that the detection of outliers using only one model is not as effective as the model designed in this paper. In particular, the detection-accuracy rate of isolated forests is only 39%, the recall rate is only 38%, and the F1 score is 0.39; the detection effect is the weakest. The outlier-detection effect of the second-layer classifier, random forest, is better than that of the isolated forest model, with an accuracy rate of 81%, a recall rate of 76%, and an F1 score of 0.72. The model proposed in this paper has the highest detection accuracy, with 96%, a recall rate of 98%, and an F1 score of 0.95. The results show that the proposed grain-and-oil-food-outlier-detection model offers excellent classification performance.

The outlier-detection model proposed in this paper has a good effect because the detection effect in the model is improved layer by layer. In the second layer of the detection model, the data with incorrect predictions in the first layer and the data with correct predictions are reconstituted as a new training set in a ratio of 1:1. The detection effect of each layer in the model is shown in Table 7.

It can be seen in Table 7 that the indicators of the first-layer classifier of the model are consistent with those of the isolated forest alone, while the second layer of the model is significantly improved compared with the random forest alone, with the accuracy rate increased by 11%, the recall rate increased by 14%, and the F1 score reaching 0.89. The detection performance of the second-layer classifier is an important reason for the excellent detection performance of this model. The third-level decision maker improves the accuracy to 96% and the recall rate to 98%. The model has the highest F1 score, indicating that it has the best classification performance.

**Figure 7:**
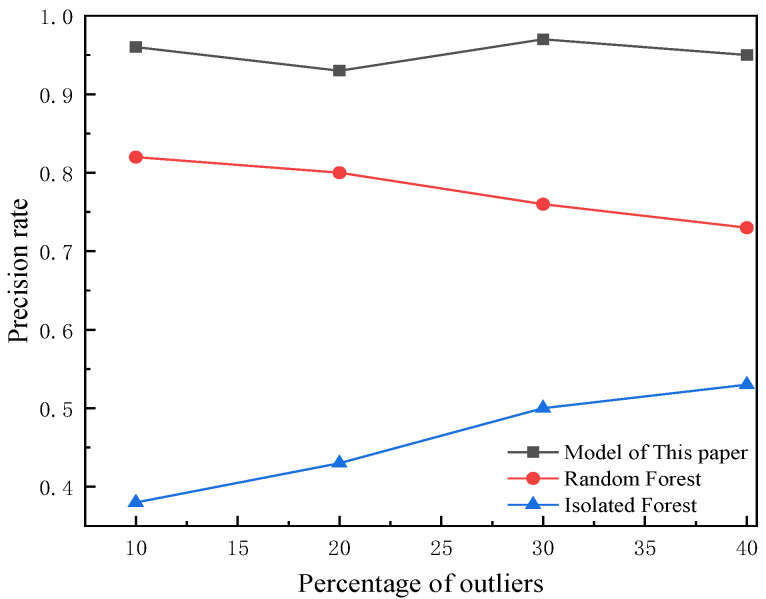
Variation in precision rates of different proportional outliers.

**Figure 8:**
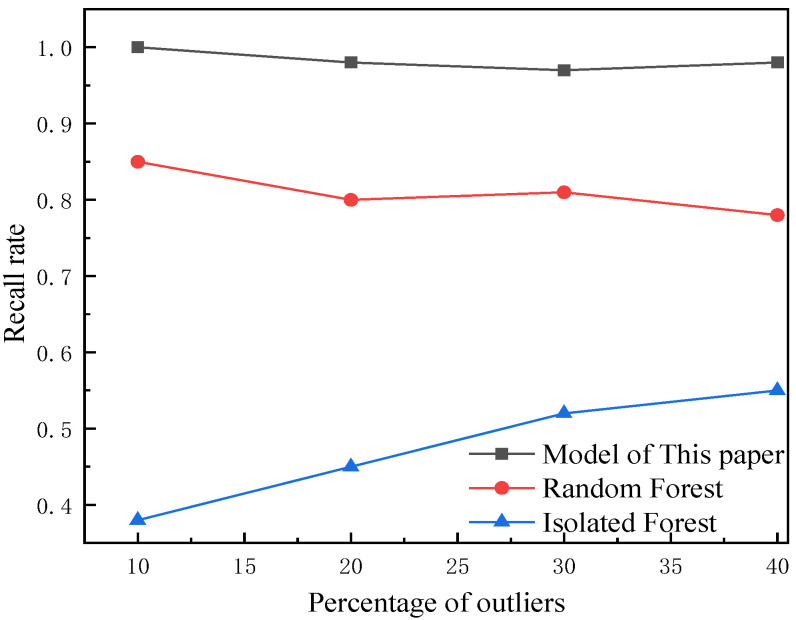
Changes in recall rate for different percentages of outliers.

The proportion of outliers in the model accounts for 10%, and different proportions of outliers are set to verify the effectiveness of the model, as shown in Figure 7 and Figure 8. With the increasing proportion of outliers, the accuracy and recall of the proposed model tend to balance, further verifying the effectiveness of the model.

In order to compare the high accuracy and recall rate of the proposed model with the traditional outlier-detection model, this study applies control models, such as the principal component analysis (PCA) algorithm, the salient object detection (SOD) algorithm, support vector machine, and AdaBoost algorithm. The PCA algorithm maps data to low-dimensional feature space by means of a data-transformation method, and then recognizes outlier. The SOD algorithm is similar to the PCA algorithm, which also uses data transformation to identify outliers. The support-vector-machine algorithm can be understood as finding a hyperplane to circle the normal data in a data set, while those outside the circle are outliers. The AdaBoost algorithm is an ensemble-learning algorithm. Its principle is that after multiple weak classifiers are trained, a strong classifier is eventually obtained for classification prediction, which is similar to the idea of this model architecture. Using the same data set, this paper compares the accuracy, recall, and F1 scores of these outlier-detection models and their different detection effects, as shown in Table 8.

In the table, we can see that although the control model established by this model can reach 70–90% in the detection accuracy of outliers, the recall rate of the model is low, and the F1 score is not high. The accuracy of the model detection in this article reached 96%, and it had the highest recall rate and F1 score when tested on the same dataset.

### 3.3. Data On-Chain Storage and Recovery Mechanism

This paper reports the use of the SM3 encryption algorithm mixed with encryption to encrypt private data before uploading them. The data-uploading algorithm is shown in Algorithm 1. Firstly, the blockchain node invokes a smart contract to initiate a transaction. Secondly, the sorting node receives the transaction information and creates the block. Finally, the master node synchronously broadcasts to other nodes. The Kafka consensus mechanism, which good scalability, was adopted in our model [37].
**Algorithm 1:** Data-upload algorithm1: if id==True then 2: priK=getKey 3: pubK=priK.PubKey 4: SM3EncryPri =SM3Encrypt(priData,pubK) 5: traceobj=traceObj(pubData,SM3EncryPri) 6: jsonfJ=SON(traceobj) 7: err= putState(jsonf) 8:   if err! ==Null then 9:     return Error 10:  else 11:    return TxID.blockNum 12: else 13:  return dataError

**Figure 9:**
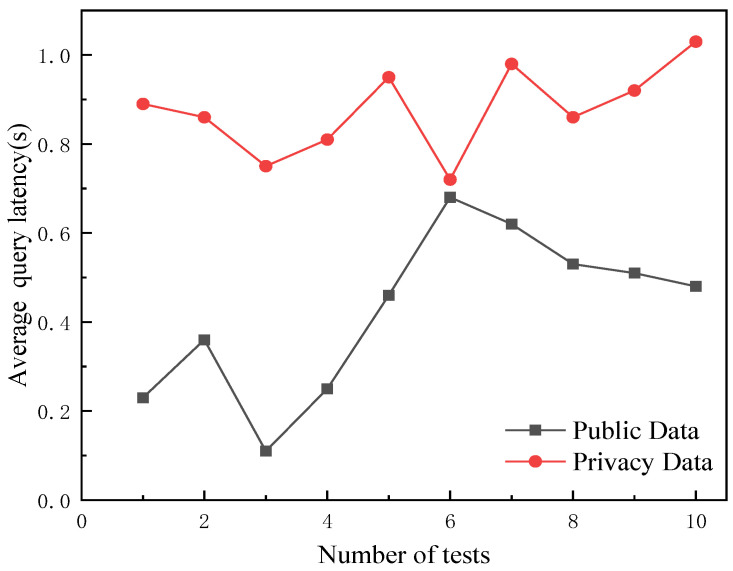
Average latency of data queries.

The traceability-information query of the grain-and-oil-food-supply chain can be divided into public-data queries and authorized queries relating to private data. Many methods are used to perform data queries, which can be undertaken based on the mapping relationship established between blockchain and local data, or through the hash value returned by IPFS. The query algorithm is shown in Algorithm 2. The ciphertext of the traceability of private data is obtained when the traceability code and SM3 private key are valid. By establishing a mapping relationship between blockchain and databases or IPFS, the data-query time is reduced. The IPFS query method for data queries was tested 10 times, with 10 queries per round and the average latency taken. In particular, the average latency for the public data queries was 0.42 s, while the average latency for the private data queries was 0.88 s, which can meet the needs of grain-and-oil-food-supply-chain-data queries. The average latencies required for public and private data queries are shown in Figure 9.
**Algorithm 2:** Data query algorithm1: if hash==True and code==True and key==True then 2:    traceinfo= Getstate(code) 3:    priEncryData=string(traceinfo.TraceData) 4:    priData=SM3Decrypt(priEncryData,key) 5:    return success 6: else 7:    return error

**Figure 10:**
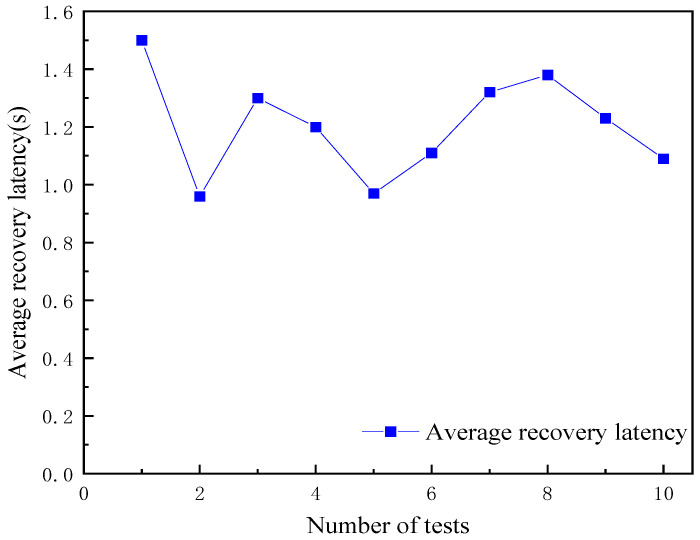
Average IPFS-data-recovery latency.

In response to the challenges faced by the blockchain based grain-and-oil-food-supply chain in terms of traceability-data storage, one of the focuses of this article is the use of “blockchain + database + IPFS” to enhance the storage capacity of the grain-and-oil-food-blockchain-traceability network. By uploading public and private data from various links in the supply chain and then storing the blockchain blocks into IPFS, the blockchain network only stores the hash values returned by IPFS, greatly alleviating the storage pressure on the blockchain-traceability network. In order to ensure the long-term storage of massive traceability data in various links in the grain-and-oil-food-supply chain, private-data protection can also be achieved. In addition, to address the potential issue of lost or damaged traceability data, this article proposes a blockchain-based data-recovery mechanism based on alleviating the storage pressure on the blockchain traceability network. By establishing a mapping relationship between blockchain and local databases, as well as a hash value between blockchain and IPFS, a connection can be established between the three. If local-database or IPFS data are lost or damaged, they can be recovered in a timely manner through this mechanism, to improve the efficiency of supply-chain traceability and the fault tolerance of grain-and-oil-food-traceability systems. The average latency of data recovery through IPFS is shown in Figure 10.

Compared with other grain-and-oil-food-traceability systems, our work focuses more closely on the authenticity of source data, and the storage pressure of blockchain is reduced. Before the data were chained, abnormal values were filtered to reduce data-storage redundancy. The accuracy rate of the proposed outlier-detection model is 96%, the recall rate is 98%, and the detection performance is excellent. In terms of data storage, the storage model and data-recovery mechanism are built by combining blockchain, the database, and IPFS, which can effectively solve the problem of tracing data loss, and the average delay in the data recovery is 1.2 s. The system’s efficiency was improved to meet the practical requirements of the grain-and-oil-food-supply chain.

## 4. Conclusions

This paper proposes a grain-and-oil-food-supply-chain-traceability model based on blockchain. Firstly, in order to ensure the accuracy of the data collected by IoT devices in the supply chain, we built an outlier-detection model. Through an experimental analysis, this model was found to offer excellent outlier detection. The accuracy and recall of the model were more than 96%, and the average score of F1 was 0.95. The results show that the model can ensure the authenticity and reliability of source information. In addition, in order to improve the storage performance of blockchain-traceability networks, a storage model called “blockchain+ database +IPFS” was introduced. With this model, the blockchain traceability network only needs to store the hash values returned by IPFS, greatly reducing the storage pressure. By establishing a data-recovery mechanism, the timely recovery of data in the event of loss or damage is ensured, and the system’s fault tolerance is ensured. The average query latency is 0.42 s for public data and 0.88 s for private data, and the average data-recovery latency was 1.2 s. Finally, a blockchain-based grain-and-oil-food-supply-chain-traceability system was designed and built using Hyperledger Fabric. The constructed model and system realized multi-source heterogeneous data uploading, lightweight storage, and data recovery in the supply chain, making the traceability data more authentic and reliable, providing a useful reference for innovative applications in the grain-and-oil-food-supply chain.

The application of blockchain technology in the grain-and-oil-food-supply-chain system has become a research focus in recent years, providing a flexible solution in the field of grain-and-oil-food traceability. However, the grain-and-oil-food-supply chain is a complex structure, and the its combination with blockchain technology still presents some challenges. In the future, we will place a greater emphasis on research into the integration of the grain-and-oil-food blockchain with IoT and artificial intelligence.

## Figures and Tables

**Table 1:** Grain-and-oil-food-supply-chain-traceability information.

Supply-Chain Stage	Acquisition Equipment	Public Data	Private Data
Planting Stage	Sensors, RFID, pesticide detectors, cameras	Grain type, origin, batch number, growing environment, growing cycle, fertilizer application, operator information, testing report, mycotoxin content, heavy-metal content, pests	Food production, pesticide residues
Processing Stage	Sensors, RFID, camera, question and answer (QA), quick response (QR) code	Processing of personnel information, grade information, quality-inspection report, entry time, factory time, operator information, mycotoxin content, heavy-metal content, pests, other hazards	Processing monitoring, processing-equipment status, grain pests
Logistic Stage	Sensors, RFID, Geographic Information System (GIS), cameras, QR codes	Logistics provider, logistics-bill number, transportation mode, driver’s name, departure place, departure time, destination, arrival time, hazardous substances in transport vehicles, residues of chemical additives. physical impurities	Shipping costs, order information, shipping routes, policy information, job monitoring
Storage Stage	Sensors, RFID, cameras, QR codes	Storage enterprise, warehouse address, warehouse number, warehouse temperature, management-personnel information, physical impurities, mycotoxin content, heavy-metal content	Warehouse costs, warehouse-order information, job monitoring
Sales Stage	RFID, camera, QR code	Sales company, sales time, sales location, sales staff, selling price, operator, physical impurities, residues of chemical additives	Input price, purchase quantity, sales quantity, work monitoring

**Table 2:** Experimental environment.

Environment	Description
Development Platform	IntelliJ IDEA 2019.3.3 ×64; VS code 1.76.2
Operating System	Windows 10; Ubuntu 16.04
Blockchain Module	Hyperledger Fabric v1.2.0; nodejs v8.10.0; go v1.12
Development Languages	Java; Javascript; Go; Nodejs
Development Framework	Spring boot; Vue

**Table 3:** Design of smart contracts.

Contracts Function	Business Logic	Methods	Descriptions
Data Upload	Public Data	AddPubData	Uploading formatted data
	Private Data	AddPriData	Encryption of data and upload of private information in cipher text
Access Control	Participating Subject	SubjectAcc	Access rights, according to the nature of the business
	Regulators	RegulatoryAcc	Regulators gain access by decrypting uploaded private data
Data Query	Public Data	TracePubData	Consumers have access to publicly available traceability data
	Private Data	TracePriData	Supervisors can decrypt uploaded private-data ciphertext

**Table 4:** Binary confusion matrix.

Actual/Predicted	Anomalies	Normal
Normal	TP	TN
Abnormal	FP	TN

**Table 5:** Evaluation metrics.

Metrics	Formulas
Precision	TP/(TP + FP)
Recall	TP/(TP + FN)
F1-score	(2 × Precision × Recall)/(Precision + Recall)

**Table 6:** Comparison of isolated forest, random forest, and this paper’s anomaly-detection framework.

Models	Precision	Recall	F1 Score
Isolated Forests	0.39	0.38	0.39
Random Forest	0.81	0.76	0.72
Model in this Paper	0.96	0.98	0.95

**Table 7:** Comparison of the detection effects of each layer of the model.

Models	Precision	Recall	F1 Score
First Layer	0.39	0.38	0.39
Second Layer	0.92	0.90	0.89
Third Layer	0.96	0.98	0.95

**Table 8:** Evaluation of the different models.

Models	Precision	Recall	F1 Score
PCA	0.87	0.17	0.20
SOD	0.73	0.62	0.31
SVM	0.88	0.65	0.53
AdaBoost	0.90	0.71	0.69
Model in this Paper	0.96	0.98	0.95

## Data Availability

Data are contained within the article.

## References

[1] Wang L., Xiao Y., Ouyang Z. (2021). Food and Grain Consumption Per Capita in the Qinghai-Tibet Plateau and Implications for Conservation. Nutrients.

[2] Liu F., Xiao X., Qin Y., Yan H., Huang J., Wu X., Zhang Y., Zou Z., Doughty R.B. (2022). Large spatial variation and stagnation of cropland gross primary production increases the challenges of sustainable grain production and food security in China. Sci. Total Environ..

[3] Chung S., Hwang J.-T., Park S.-H. (2022). Physiological Effects of Bioactive Compounds Derived from Whole Grains on Cardiovascular and Metabolic Diseases. Appl. Sci..

[4] Xu Y., Li J., Zhao J., Wang W., Griffin J., Li Y., Bean S., Tilley M., Wang D. (2020). Hempseed as a nutritious and healthy human food or animal feed source: A review. Int. J. Food Sci. Technol..

[5] Chen C., Yang B., Gao A., Li L., Dong X., Zhao F.-J. (2022). Suppression of methanogenesis in paddy soil increases dimethylarsenate accumulation and the incidence of straighthead disease in rice. Soil Biol. Biochem..

[6] Malik M., Mahmood S., Noreen S., Abid R., Ghaffar S., Zahra S., Shah T., Ahmad A. (2021). Lead contamination affects the primary productivity traits, biosynthesis of macromolecules and distribution of metal in durum wheat (*Triticumdurum* L.). Saudi J. Biol. Sci..

[7] Al-Saidi M., Hussein H. (2021). The water-energy-food nexus and COVID-19: Towards a systematization of impacts and responses. Sci. Total Environ..

[8] Liu P., Long Y., Song H.-C., He Y.-D. (2020). Investment decision and coordination of green agri-food supply chain considering information service based on blockchain and big data. J. Clean. Prod..

[9] Erukainure F.E., Parque V., Hassan M.A., FathEl-Bab A.M.R. (2022). Estimating the stiffness of kiwifruit based on the fusion of instantaneous tactile sensor data and machine learning schemes. Comput. Electron. Agric..

[10] Jiang Y., Zheng W. (2021). Coupling mechanism of green building industry innovation ecosystem based on blockchain smart city. J. Clean. Prod..

[11] Mahmudnia D., Arashpour M., Yang R. (2022). Blockchain in construction management: Applications, advantages and limitations. Autom. Constr..

[12] Kamble S.S., Gunasekaran A., Sharma R. (2020). Modeling the blockchain enabled traceability in agriculture supply chain. Int. J. Inf. Manag..

[13] Li K., Lee J.-Y., Gharehgozli A. (2021). Blockchain in food supply chains: A literature review and synthesis analysis of platforms, benefits and challenges. Int. J. Prod. Res..

[14] Hu J., Zhu P., Qi Y., Zhu Q., Li X. (2022). A patent registration and trading system based on blockchain. Expert Syst. Appl..

[15] Qu Z., Zhang Z., Zheng M. (2022). A quantum blockchain-enabled framework for secure private electronic medical records in Internet of Medical Things. Inf. Sci..

[16] Shaikh F.K., Karim S., Zeadally S., Nebhen J. (2022). Recent Trends in Internet-of-Things-Enabled Sensor Technologies for Smart Agriculture. IEEE Internet Things J..

[17] Zhang Q., Han Y.-y., Su Z.-b., Fang J.-l., Liu Z.-q., Wang K.-y. (2020). A storage architecture for high-throughput crop breeding data based on improved blockchain technology. Comput. Electron. Agric..

[18] Peng X., Zhang X., Wang X., Li H., Xu J., Zhao Z. (2022). Construction of rice supply chain supervision model driven by blockchain smart contract. Sci. Rep..

[19] Li Y., Zhang X., Zhao Z., Xu J., Jiang Z., Yu J., Cui X. (2022). Research on Grain Food Blockchain Traceability Information Management Model Based on Master-Slave Multichain. Comput. Intell. Neurosci..

[20] Zhang G., Chen X., Feng B., Guo X., Hao X., Ren H., Dong C., Zhang Y., Chen Y. (2022). BCST-APTS: Blockchain and CP-ABE Empowered Data Supervision, Sharing, and Privacy Protection Scheme for Secure and Trusted Agricultural Product Traceability System. Secur. Commun. Netw..

[21] Violino S., Pallottino F., Sperandio G., Figorilli S., Ortenzi L., Tocci F., Vasta S., Imperi G., Costa C. (2020). A Full Technological Traceability System for Extra Virgin Olive Oil. Foods.

[22] Xu J., Han J., Qi Z., Jiang Z., Xu K., Zheng M., Zhang X. (2022). A Reliable Traceability Model for Grain and Oil Quality Safety Based on Blockchain and Industrial Internet. Sustainability.

[23] Li J., Wu J., Chen L. (2018). Block-secure: Blockchain based scheme for secure P2P cloud storage. Inf. Sci..

[24] Peng X., Zhang X., Wang X., Xu J., Li H., Zhao Z., Qi Z. (2022). A Refined Supervision Model of Rice Supply Chain Based on Multi-Blockchain. Foods.

[25] Xiong W., Xiong L. (2021). Anti-collusion data auction mechanism based on smart contract. Inf. Sci..

[26] Xu J., Zhao Y., Chen H., Deng W. (2023). ABC-GSPBFT: PBFT with grouping score mechanism and optimized consensus process for flight operation data-sharing. Inf. Sci..

[27] Lei M., Xu L., Liu T., Liu S., Sun C. (2022). Integration of Privacy Protection and Blockchain-Based Food Safety Traceability: Potential and Challenges. Foods.

[28] Feng H., Wang X., Duan Y., Zhang J., Zhang X. (2020). Applying blockchain technology to improve agri-food traceability: A review of development methods, benefits and challenges. J. Clean. Prod..

[29] Tripathi A.K., Akul Krishnan K., Pandey A.C. (2023). A Novel Blockchain and Internet of Things-Based Food Traceability System for Smart Cities. Wirel. Pers. Commun..

[30] Jiang J., Li T., Chang C., Yang C., Liao L. (2022). Fault diagnosis method for lithium-ion batteries in electric vehicles based on isolated forest algorithm. J. Energy Storage.

[31] Domingues R., Filippone M., Michiardi P., Zouaoui J. (2018). A comparative evaluation of outlier detection algorithms: Experiments and analyses. Pattern Recognit..

[32] Liu H., Zhang S., Wu X. (2014). MLSLR: Multilabel Learning via Sparse Logistic Regression. Inf. Sci..

[33] Xu Z., Shen D., Nie T., Kou Y., Yin N., Han X. (2021). A cluster-based oversampling algorithm combining SMOTE and k-means for imbalanced medical data. Inf. Sci..

[34] Gabbay I., Shapira B., Rokach L. (2021). Isolation forests and landmarking-based representations for clustering algorithm recommendation using meta-learning. Inf. Sci..

[35] Zheng X., Xu C., Hu X., Zhang Y., Xiong X. (2020). The Software/Hardware Co-Design and Implementation of SM2/3/4 Encryption/Decryption and Digital Signature System. IEEE Trans. Comput.-Aided Des. Integr. Circuits Syst..

[36] Vimal S., Srivatsa S.K. (2019). A new cluster P2P file sharing system based on IPFS and blockchain technology. J. Ambient. Intell. Humaniz. Comput..

[37] Gao N., Han D., Weng T.-H., Xia B., Li D., Castiglione A., Li K.-C. (2022). Modeling and analysis of port supply chain system based on Fabric blockchain. Comput. Ind. Eng..

